# Effect of transcranial alternating current stimulation (NET Device™) on psychostimulant withdrawal severity and time course: a real-world data analysis

**DOI:** 10.3389/fpsyt.2026.1899599

**Published:** 2026-09-24

**Authors:** Mark K. Greenwald, Lana R. Grasser, Joe R. Winston

**Affiliations:** 1 Department of Psychiatry and Behavioral Neurosciences, Wayne State University, Detroit, MI, United States; 2 Department of Psychology, College of Liberal Arts and Sciences, Wayne State University, Detroit, MI, United States; 3 Ben L. Silberstein Institute for Brain Health, Division of Research and Innovation, Wayne State University, Detroit, MI, United States; 4 NET Recovery Corp.™ (NRC), Wilmington, DE, United States

**Keywords:** ACSA, NET device, neuromodulation, psychostimulant withdrawal, real-world data, real-world evidence, stimulant use disorder, transcranial alternating current stimulation

## Abstract

**Background:**

Psychostimulant use disorder is a life-threatening condition with no FDA-approved treatment. Stimulant withdrawal symptoms impede treatment engagement and are unaddressed by standard of care. The NET Device, a non-invasive transcranial alternating current stimulator, is FDA-cleared for opioid withdrawal suppression. No studies have evaluated whether NET Device use attenuates psychostimulant withdrawal.

**Methods:**

Electronic health record and device utilization data were collected from 109 stimulant-only users and 112 stimulant/opioid co-users (*N* = 221 adults total; *M*_age_=39.2, *SD*_age_=10.7; 119M) entering residential addiction treatment (*n* = 168) or detention centers (*n* = 53). The Amphetamine Cessation Symptom Assessment (ACSA) was administered at baseline and 1 hour (day 1), and twice daily on days 1–7. A linear mixed model (LMM) with random intercepts and slopes was used to analyze the trajectory of ACSA scores.

**Results:**

ACSA severity and time course were similar across settings (residential vs. detention). A two-phase LMM separated acute (baseline to 1 hour) and post-acute withdrawal symptom reductions. Relative to baseline ACSA scores, stimulant-only and co-user groups each reported significant reductions at 1 hour (46% and 33%), day 2 morning (59% and 42%), and decelerating log-time trajectory through day 4 (79 hours), the primary analysis window. Estimates beyond day 4 rest on 6.9% of observations and are reported as exploratory only.

**Conclusion:**

NET Device monotherapy was associated with rapid reduction in psychostimulant withdrawal severity regardless of setting and opioid co-use. No device-related adverse events were reported. These real-world data support initiation of a sham-controlled randomized trial to evaluate NET Device efficacy for stimulant use disorder.

## Introduction

Psychostimulant use disorder, driven predominantly by methamphetamine and cocaine, represents one of the most urgent and inadequately addressed crises in contemporary addiction medicine. Overdose deaths involving psychostimulants increased 600% from 2012 to 2023, with over 36,000 deaths in 2023 alone, and methamphetamine is now implicated in approximately one in three overdose deaths nationally (1, 2). Stimulant use commonly co-occurs with opioid use, producing polysubstance scenarios with dramatically elevated mortality risk (3, 4). Despite the scale and trajectory of this crisis, and significant efforts to develop medications, there are no FDA-approved pharmacotherapies for stimulant use disorder (5–8), in stark contrast to opioid use disorder (methadone, buprenorphine, naltrexone), alcohol use disorder (naltrexone, acamprosate, disulfiram), and tobacco use, each of which has approved pharmacological options (9).

Acute psychostimulant withdrawal is characterized by dysphoria, fatigue, insomnia or hypersomnia, anhedonia, irritability, agitation, poor concentration, psychomotor retardation, increased appetite, and intense drug craving (10–13). Without targeted intervention, these symptoms peak within 2–3 days following discontinuation (acute phase), and show approximately 40–60% natural resolution over 7–10 days (14, 15). Several pharmacological agents have been evaluated for stimulant withdrawal suppression across settings, and recent literature reviews have uniformly concluded these agents have minimal or no efficacy (6, 16–18). In an open-label inpatient study, McGregor et al., 2008b found that patients treated with mirtazapine or modafinil reported a reduction in scores on the Amphetamine Cessation Symptom Assessment (ACSA), which was validated in this population (14), but meaningful symptom reduction was not evident until days 7–10 of treatment, with no acute benefit (19). Moreover, a contemporaneous randomized controlled trial found that mirtazapine did not suppress ACSA scores more than placebo (20). The lack of interventions for reducing stimulant withdrawal symptoms is an unmet clinical need. It is critical to intervene when symptoms are severe and patients are most vulnerable to treatment dropout and relapse, because effective management of stimulant withdrawal symptoms during this acute phase could improve retention and engagement in longer-term treatment (21).

NeuroElectric Therapy™ (NET^®^) is a transcranial alternating current stimulation (tACS) modality delivered by the NET Device, a non-invasive, battery-powered, self-administered device that applies bilateral transcutaneous ACS to the mastoid processes for up to 7 days. The NET Device received FDA 510(k) market clearance in May 2024 for use in patients experiencing opioid withdrawal symptoms (K233166) (22). In a randomized, sham-controlled pivotal trial, active NET Device stimulation produced a 61.3% reduction in opioid withdrawal severity on the Clinical Opiate Withdrawal Scale (COWS; 23) at 1 hour, substantially exceeding the pre-specified 15% superiority criterion and significantly more than sham, and the active group was significantly less likely to request medications for opioid use disorder during the inpatient period (24). Post-discharge data from the same cohort showed that participants who self-administered active stimulation >24 hours reported 3 to 5 times fewer use-days of illicit opioids or psychostimulants at 12 weeks (25).

Whether the NET Device’s effects on withdrawal extend to psychostimulant withdrawal is not established. Mechanistically, stimulant withdrawal involves monoaminergic (especially dopaminergic but also serotonergic and adrenergic) deficit and dysregulation of mesolimbic reward circuitry (26, 27), pathways that partially overlap with opioid withdrawal neurobiology. The NET Device’s proposed mechanism involves modulation of endogenous opioid and dopaminergic systems through extended transcranial electrical stimulation of the mastoid region (28, 29). The ACSA and COWS instruments share overlapping withdrawal symptom domains (anxiety, restlessness, sweating, gastrointestinal discomfort), though the ACSA more specifically captures the post-acute ‘crash’ features of stimulant cessation: fatigue, hypersomnia, increased appetite, vivid dreams, and craving (30).

The present study reports a real-world data (RWD) analysis of NET Device effects on psychostimulant withdrawal severity and time course. Stimulant withdrawal symptom data were collected with the ACSA prospectively during routine clinical operations of NET Recovery Corp.’s treatment program from adults entering residential addiction treatment or detention centers. Aims were to: (i) determine whether NET Device use is associated with clinically meaningful reduction in psychostimulant withdrawal severity during the acute treatment period; (ii) characterize the time course of symptom reduction; and (iii) visualize the reduction in stimulant withdrawal symptoms relative to published data from a medication study using the ACSA (19). Long-term substance use outcomes are not addressed in this report.

## Methods

### Study design and settings

This study constitutes a real-world data (RWD) analysis as defined under 21st Century Cures Act Section 3022 (31) and FDA’s real-world evidence (RWE) Framework (32). RWD were collected prospectively during routine clinical operations of NET Recovery Corp.’s Kentucky treatment program during calendar year 2025. No experimental manipulation, randomization, or protocol-driven intervention was imposed; all participants received the NET Device open-label as part of standard clinical care, with prospective observational assessments.

Participating facilities comprised seven residential addiction treatment facilities and five detention centers in Kentucky. IRB review was not required for this analysis of de-identified data (see Ethics Statement).

Data sources included electronic health records and patient-generated device utilization data captured as standard practice across participating facilities. Analysis of these RWD constitutes RWE regarding the association of device utilization/stimulation with acute psychostimulant withdrawal suppression under routine treatment conditions.

### Participants

Participants were *N* = 221 adults (≥18 years; *M*_age_=39.2 years, *SD*_age_=10.7; 119M) who presented to a participating facility (*n* = 168 enrolled at residential treatment facilities and *n* = 53 enrolled at detention centers). Adult stimulant-only (*n* = 109) and stimulant/opioid co-users (*n* = 112) were eligible if they screened positive for psychostimulants at admission by urine drug screen (UDS) or reported prior-month psychostimulant use at intake (whereas it was not feasible to obtain a reliable diagnosis of Stimulant Use Disorder in these facilities), and initiated NET Device use as part of routine clinical care at each facility. Exclusion criteria were: presence of an implanted electronic device (e.g., pacemaker, implantable cardioverter-defibrillator, deep brain stimulator), history of epilepsy, and – because the NET Device is intended as monotherapy during detoxification – concurrent opioid agonist therapy (methadone or buprenorphine). Participants were not excluded based on concurrent illicit opioid use. For stimulants and opioids, there was no washout period (i.e. no delay from last use of the substance to beginning of device exposure). Concurrent sedative or alcohol withdrawal was not exclusionary; based on clinician judgment, participants with these presentations were tapered using adjunctive medications, which were then discontinued, and then offered the NET Device. No minimum or maximum stimulant withdrawal symptom severity threshold was applied. Group assignment was determined by the admission urine drug screen: participants whose screen was negative for opioids were classified as stimulant-only, and those positive for one or more opioids as stimulant/opioid co-users. Assignment therefore reflects opioid exposure detectable at screening rather than self-reported opioid history (see **Table 1**).

**Table 1:** Quantitative and temporal characteristics of psychostimulant use, by group.

Characteristic	Stimulant-only	Stimulant/opioid co-user
*n*	109	112
Any opioid use documented at intake (MIA), *n*	39/109	108/112
Primary stimulant, *n*		
Methamphetamine	81	83
Cocaine/crack	22	21
Amphetamine/other/unclear	6	8
Daily/near-daily use, *n*	66/93	73/104
Days since last use, median (IQR)	4 (2–7)	3 (2–7)
Last use within 7 days, *n*	70/93	76/97
Recency recorded, *n*	93	97

IQR, interquartile range. All characteristics in this table are abstracted from the Medical Intake Assessment (MIA) completed by the treatment facility at admission; group assignment derives from the admission urine drug screen and is therefore not interchangeable with the MIA opioid field. The MIA records opioid involvement of any recency, from current use through lifetime history. Among the 39 stimulant-only participants with opioid use noted at intake, the most recent recorded use was more than 12 months before admission for 11, within the preceding 12 months for 17 (11 of those within 30 days), and not resolvable from the record for 11. Denominators differ by row because not every intake recorded every field, and two participants (both stimulant/opioid co-users) had no MIA available. Days since last use computed relative to the screening date. Amount was recorded in semi-quantitative clinical terms and is not tabulated.

The device was offered by clinical staff at each participating facility to all patients meeting the stimulant-positive criterion and none of the exclusion criteria during routine intake; no additional clinical gatekeeping was applied.

### Intervention

The NET Device delivers tACS via transcutaneous, gel-filled electrodes (approximately 1cm x 2cm) attached bilaterally to the mastoid processes (M1 and M2 electrode locations, adjacent to T7 and T8 electrode locations, respectively, in the 10/10 EEG system). The device is non-invasive, battery-powered, portable (pocket sized), and re-usable.

The device delivers multiple low-amperage waveforms at controlled frequencies and pulse widths that vary throughout each treatment day, with no net direct current component. In prior clinical studies, the output waveform was optimized for dynamic variations in skin impedance, electrode conductance, frequency and pulse-width related sensation, and orthogonal electrode pressure (e.g., from head pressure when sleeping), leading to improved rates of patient tolerability. Stimulation output frequency varied from 4 to 3000 Hz and pulse width from 7 to 750 microseconds. Stimulation output current varied from 0 to 3.2 mA (peak) into a 15 kOhm load, and output voltage varied from 0 to 44 volts (peak to peak).

Human and animal studies, and clinical observation, have identified different electronic waveforms corresponding to subtypes of polysubstance use. These waveforms were programmed into the device at treatment entry based on each participant’s UDS results at the time of treatment initiation. For stimulant-only users, waveforms were programmed to be stimulant-specific. For stimulant + opioid polysubstance users, waveforms were programmed on day 1 to be more opioid-specific because opioid withdrawal symptoms are more tied to the patient’s initial distress; thus, we treated those symptoms first; on day 2, waveforms were added to target stimulant withdrawal symptoms, which take longer to emerge and are relatively less distressing.

Stimulation was continuously available (except when bathing) for up to 7 days. Treatment was self-administered on an “as needed” basis (i.e. no scheduled/mandatory requirements), and participants were instructed that they could control the device output intensity and duration according to perceived benefit. Training in self-administration of device stimulation was provided to all participants using a standardized protocol administered by the Sponsor’s technical staff at each facility. Staff reviewed the typical stimulation experience, device user interface (knob and indicator LEDs and battery), methods of electrode attachment and (if necessary) reattachment in case of unintentional disconnection or showering, and self-administration of stimulation intensity and duration according to perceived benefit. The device logs intensity at 5-min intervals during the entire period of stimulation. Patients’ perceived benefit is measured in two ways: (i) by relative increase or decrease in the intensity knob setting (i.e. an increase in knob intensity usually corresponds to a desire to reduce withdrawal symptoms, whereas a decrease in knob intensity usually corresponds to greater wellbeing), and (ii) the duration of stimulation the patient chooses (i.e. when the patient turns the intensity to a low level [<15%] or off, indicating no further perceived benefit).

No pharmacological agent was administered as a primary withdrawal treatment. Only during the period of device stimulation, patients were not allowed to use MOUD (buprenorphine, methadone or naltrexone), gabapentinoids, tricyclic antidepressants, dextromethorphan, certain sleep medications (e.g. trazodone, doxylamine, hydroxyzine, diphenhydramine), antipsychotics, pseudoephedrine, CNS-acting antihypertensives (e.g. clonidine, lofexidine) and beta blockers. Importantly, once device stimulation stopped, these medications could be used again. Concurrent psychosocial care, group therapy, individual counseling, and any adjunctive symptomatic medications (e.g., over-the-counter analgesics, antiemetics) reflected routine care, which varied substantially across facility types. Behavioral health interventions differed between addiction treatment and detention centers. However, medication treatments were comparable across facilities/settings. Any differences in care were a function of site-specific medical practices and preferences. Residential addiction treatment centers typically provide structured group and individual counseling, while detention centers provide minimal behavioral support beyond basic medical care. All psychosocial treatments were primarily delivered following NET Device use (i.e. after the first few residential days), so those treatments should not confound the stimulant withdrawal data presented here. Facility-level concurrent-care details were not collected at the participant level for this analysis.

### Outcome measure

Psychostimulant withdrawal severity was quantified using the Amphetamine Cessation Symptom Assessment (ACSA), a validated 16-item self-report instrument scored 0 (not at all) to 4 (extremely) per item, yielding a total score range of 0–64 (14). The ACSA has a few questions that may reflect shared opioid and stimulant withdrawal symptoms (e.g. hypersomnia, irritability, anxiety), thus, scores may partly decrease together. The ACSA was administered at baseline (immediately prior to initiating device use), at 1-hour post-initiation, and twice daily on days 1–7 while the participant used the device; data collection ceased after device stoppage. Cronbach’s alpha=0.84 indicated good reliability in our sample. Intraclass correlation coefficients reported in the **Supplementary Materials** support test-retest reliability while also acknowledging anticipated change as this is the primary outcome variable in the present study measuring withdrawal severity.

### Reference comparator

For contextual comparison of the speed and magnitude of withdrawal reduction, ACSA scores from this study are referenced against ACSA scores in a study by McGregor et al. (19), who used a non-randomized design to evaluate stimulant withdrawal suppression by mirtazapine (titrated to 60 mg/day; *n* = 13) vs. modafinil (200 mg BID; *n* = 14) vs. pericyazine (treatment as usual; *n* = 22), with adjunctive medications as needed (e.g., benzodiazepines for anxiety and insomnia, non-opioid analgesia), in adults undergoing methamphetamine detoxification over 10 days in an inpatient setting. None of the three medications used in the McGregor et al. study (mirtazapine, modafinil, pericyazine) were used in the present study. As noted earlier, the use of adjunctive medications in the present study was restricted during device use.

The ACSA version used in the present study is identical in scoring range (0–64) to the version used by McGregor et al. (19), permitting comparison of raw scores between datasets. However, this comparison is strictly descriptive: the two datasets differ in design, population, and treatment conditions.

### Safety assessment

We used a structured questionnaire to assess device-related adverse events (AEs) each time the patient was interviewed, typically 1–2 times daily for the duration of device use.

### Statistical analysis

Prior to primary modeling, we first assessed the pattern of missing data, and the potential effects of setting (residential vs. detention) and stimulant group (stimulant-only vs. stimulant/opioid co-user) as sources of heterogeneity in ACSA trajectory. We also computed descriptive statistics for baseline demographic and clinical characteristics of the sample (mean + SD for continuous variables, percentages for categorical variables); we then examined whether these characteristics differed by setting or stimulant group (using Welch’s t-test for continuous variables, or Pearson’s chi-square with Yates continuity correction for categorical variables).

Participants with fewer post-baseline observations had significantly higher baseline ACSA scores than those with more complete data (mean 33.5 vs. 27.4, *p* = .017) and shorter device use duration (mean 9.9 vs. 44.1 hours, *p* <.001). This pattern is consistent with missing at random (MAR) rather than missing completely at random (MCAR): missingness was systematically related to observed baseline covariates rather than being random with respect to all data. This was supported by the Little’s MCAR test that provided evidence against MCAR, *χ*^2^(713)=115874.45, *p* <.001. Complete-case analysis (e.g., repeated-measures ANOVA requiring complete data across a restricted window) would therefore produce biased estimates by selectively retaining participants with lower baseline severity and longer device use. The linear mixed model (LMM) framework, which uses all available observations per participant under the MAR assumption via maximum likelihood estimation, was selected as the primary analytic approach. Standardized metrics of effect size and overall model fit are reported using an established approach (33).

#### Rationale for the MAR assumption

Two end-of-stimulation mechanisms were considered. First, patients who experienced sufficient withdrawal symptom reduction to feel improved may have self-discontinued device use and stopped reporting (*success*-driven end of stimulation). Second, patients who felt the device was not providing adequate benefit may have discontinued and stopped reporting (*failure*-driven end of stimulation). Under both mechanisms, the patient’s decision to end their device stimulation is predicted by their observed prior ACSA trajectory: declining scores precede success-driven end of stimulation, and persistently elevated scores precede failure-driven end of stimulation. Because the probability of ending stimulation is predictable from observed data in both cases, these mechanisms are consistent with MAR rather than missing not at random (MNAR). The LMM, fit under MAR via maximum likelihood, uses all available observations and produces valid inference under these conditions. The two mechanisms generate biases in opposite directions at late timepoints: success-driven end of stimulation biases observed means upward (higher-severity patients overrepresented among those still being measured), while failure-driven end of stimulation biases them downward (lower-severity patients overrepresented). Observed-minus-predicted residuals at days 3–4 are modestly negative (−1 to −3.5 points), consistent with a slight net failure-driven pattern, but neither mechanism dominates and the LMM estimates are stable across the primary assessment window.

Each ACSA assessment was timestamped using a uniform facility schedule: the admission day is designated day 1, with the baseline assessment at 9am, the 1-hour post-initiation assessment at 10am (which constitutes the day 1 morning assessment), and subsequent morning and afternoon assessments at 10am and 4pm respectively. A per-participant timestamp file was not available; the uniform schedule represents the protocol-specified assessment window at each facility.

The primary analysis used a two-phase LMM with the following fixed effects: (i) a post-device indicator (coded 0 at baseline and 1 at all post-baseline timepoints), representing the acute change associated with device activation; (ii) log-transformed hours since device activation (log[hours_since_device + 0.5], set to 0 at baseline by construction), representing the post-acute decelerating decline; (iii) group (coded 1 for stimulant-only users and 0 for stimulant/opioid co-users as the reference); and (iv) two-way interactions between group and each time term. The model included participant-specific random intercepts and random slopes on log-time.

The primary model used the 1,236 observations (93.1% of the 1,328 available) falling within the primary analysis window from 221 participants (median 5 observations per participant, IQR 4–8, range 1–15), estimated by restricted maximum likelihood (REML) in Python (statsmodels version 0.14). The primary analysis window was baseline through the day 4 afternoon assessment (79 hours), the last assessment falling within the interval over which both groups retained substantive coverage. Assessments from day 5 onward (92 observations, 6.9% of the total) were excluded from the primary model and analyzed only in a secondary exploratory fit reported in the **Supplementary Materials**. Descriptive statistics (mean, SD, and n with available data) are reported for each ACSA assessment timepoint through day 7. Observed means at timepoints outside the primary analysis window are presented for completeness but interpreted with caution given sparse coverage.

#### Rationale for the two-phase specification

A single smooth function of log-time cannot simultaneously represent the discrete transition in withdrawal symptom severity from device-off (baseline) to device-on (1 hour post-stimulation) and the continued decline in symptoms during ongoing device use. On the log-time scale, baseline and the 1-hour timepoint occupy adjacent positions (log[0.5]=−0.69, log[1.5]=+0.41), so a single-slope log-time model is mathematically constrained to predict only a small ACSA change between these two assessments. The observed baseline-to-1-hour change is approximately 11 points; a single-log-time model misfits this by approximately ±5 points and cannot fit the acute reduction without also degrading fit across the remaining trajectory. The two-phase specification resolves this by representing the device stimulation effect with a dedicated off/on indicator and the subsequent decline with a continuous log-time term. Model comparison supported this specification: the two-phase model had substantially better Akaike Information Criterion (ΔAIC=127.9) and Bayesian Information Criterion (ΔBIC=117.7) than a single-log-time specification. A sensitivity analysis adding an AM/PM time-of-day indicator did not improve fit (likelihood-ratio χ²[1]=2.84, *p* = .092). The AM/PM contrast did not reach significance, but it is not negligible, and within-day variation should not be described as fully absorbed by the log-time term. Information criteria and likelihood-ratio tests were computed from maximum-likelihood fits; reported coefficients are from REML fits.

By way of sensitivity analysis, in the **Supplementary Materials** we report iterative model testing with time continuously modeled equivalently over the 8 assessment points.

#### Pattern-mixture sensitivity analysis

To quantify the robustness of primary findings to departures from MAR, a pattern-mixture sensitivity analysis was performed. For each missing participant-timepoint cell, imputed ACSA values were generated as the MAR-based prediction (individualized by participant random-effect BLUPs) plus a fixed shift delta and residual noise drawn from the fitted residual distribution. Positive delta represents failure-driven MNAR, in which participants who ended stimulation early are assumed to have higher unobserved ACSA scores than MAR predicts. Ten multiple imputations were generated for each delta ∈ {0, 5, 10, 15, 20}; the LMM was refit on each completed dataset, and estimates were pooled across imputations using Rubin’s rules. The delta=0 condition served as validation (reproducing the primary MAR fit). The analysis was conducted in Python using statsmodels 0.14 and NumPy.

## Results

### Sample characteristics

The analytic sample comprised *N* = 221 participants (119 male, 102 female; mean age 39.2 years, SD 10.7, range 19–70). Race was 85.1% white/Caucasian, 9.0% Black, and 0.9% multiracial or other race, and 5% missing. Of the sample, 109 (49%) were stimulant-only users and 112 (51%) were stimulant/opioid co-users, defined by UDS or self-reported prior-month use at admission. Participants were enrolled at residential treatment facilities (*n* = 168, 76%) and detention centers (*n* = 53, 24%). Mean device use duration was 40.42 hours (SD 30.96; range 0–172.5 hours). Demographic and clinical characteristics did not differ significantly by stimulant group or by setting. Full sample characteristics are presented in **Table 2**.

**Table 2:** Baseline demographic and clinical characteristics of the analytic sample.

Characteristic	All (N = 221)	Stimulant-only (n=109)	Stimulant/opioid co-user (n=112)	Test statistic	*P*
Age, years, mean (SD)	39.2 (10.7)	39.5 (12.0)	38.8 (9.4)	-0.46	.647
Sex					
Male, *n* (%)	119 (53.8%)	63 (57.8%)	56 (50.0%)	1.06	.304
Female, *n* (%)	102 (46.2%)	46 (42.2%)	56 (50.0%)		
Race					
White, *n* (%)	188 (85.1%)	84 (77.1%)	104 (92.9%)	10.21	.001
Black, *n* (%)	20 (9.0%)	17 (15.6%)	3 (2.7%)		
Other or >1 race, *n* (%)	2 (0.9%)	1 (0.9%)	1 (0.9%)		
Not recorded, *n* (%)	11 (5.0%)	7 (6.4%)	4 (3.6%)		
Setting					
Residential, *n* (%)	168 (76.0%)	88 (80.7%)	80 (71.4%)	2.14	.144
Detention, *n* (%)	53 (24.0%)	21 (19.3%)	32 (28.6%)		
Device use duration, hours, mean (SD)	40.42 (30.96)	36.53 (27.33)	44.20 (33.81)	1.86	.065
Baseline ACSA (0–64), mean (SD)	28.1 (11.9)	25.6 (11.1)	30.4 (12.2)	3.01	.003

Data are *n* (%) for categorical variables and mean (SD) for continuous variables. Group comparisons: Welch’s *t*-test for continuous variables; Pearson χ² with Yates continuity correction for all categorical variables. “Residential” treatment includes Treatment Center, Drug Treatment Court, and Treatment Event facility types; “Detention” center is coded as its own category. *p*, *p*-value for between-group (stimulant-only vs. stimulant/opioid co-user) comparison; “—” indicates *p*-value reported on an earlier row of the same contrast. The race comparison contrasts White vs. Black only; the 2 participants recorded as other or more than one race and the 11 with race not recorded were excluded from that test. SD, standard deviation.

Daily or near-daily psychostimulant use was reported by the majority of participants for whom frequency was recorded (see **Table 1** for use characteristics). Across the cohort, median time from last psychostimulant use to treatment start was 4 days (IQR 2–7; *n* = 191). Most participants presented in the subacute window: 77% reported last use within 7 days and 87% within 14 days of treatment start. A small minority (7 of the 191 participants with a recorded last-use date, 4%) reported more than 30 days since last stimulant use; however, all were UDS-positive for stimulants at the admission screen thus meeting the stimulant-use inclusion criterion. Recency was comparable across groups (stimulant-only median 4 days, IQR 2–7; stimulant/opioid co-user median 3 days, IQR 2–7).

### Setting equivalence

ACSA severity and time course did not differ significantly between residential treatment (*n* = 168) and detention center (*n* = 53) subgroups (all *p*s>.05). These setting groups were combined for all subsequent analyses. Sensitivity analysis reported in the **Supplementary Materials** supported this analytic decision, given a lack of non-independence of observations by facility type.

### Magnitude of withdrawal symptom reduction

Observed ACSA means by timepoint and subgroup are presented in **Table 3**; descriptive statistics and acute change in individual withdrawal symptoms from baseline to 1 hour post-stimulation are reported in **Table 4**. At baseline, combined-sample mean ACSA was 28.1 (SD 11.9); stimulant-only users scored 25.6 (SD 11.1) and stimulant/opioid co-users scored 30.4 (SD 12.2). By 1 hour, the combined mean had fallen to 17.1 (SD 10.8): 13.8 (SD 10.4) in stimulant-only users and 20.3 (SD 10.3) in stimulant/opioid co-users, representing observed reductions of 46% and 33% from respective baselines. By day 2 morning, means were 10.4 (SD 10.2) for stimulant-only and 17.5 (SD 11.0) for stimulant/opioid co-users, representing observed reductions of 59% and 42%. The between-group difference in observed means was consistent across all timepoints within the primary analysis window, where sample sizes support reliable description (at the final assessment in the window, stimulant-only *n* = 23, stimulant/opioid *n* = 30). Observed means at timepoints outside the primary analysis window are presented descriptively only. The random intercepts model from the sensitivity analysis described in the **Supplementary Materials** corroborates the mean group differences in withdrawal severity, with stimulant/opioid users reporting greater severity.

**Table 3:** Observed ACSA total scores at each assessment timepoint, by subgroup.

Timepoint	Stimulant-only *n*/mean (SD)	Stimulant/opioid co-user *n*/mean (SD)	Combined *n*/mean (SD)
Baseline	109/25.6 (11.1)	112/30.4 (12.2)	221/28.1 (11.9)
1 hour	102/13.8 (10.4)	106/20.3 (10.3)	208/17.1 (10.8)
Day 1 (PM)	85/12.0 (10.3)	65/17.3 (9.9)	150/14.3 (10.4)
Day 2 (AM)	97/10.4 (10.2)	80/17.5 (11.0)	177/13.6 (11.1)
Day 2 (PM)	79/7.9 (9.3)	69/14.6 (10.0)	148/11.0 (10.2)
Day 3 (AM)	60/7.2 (8.2)	66/13.6 (10.3)	126/10.6 (9.9)
Day 3 (PM)	46/6.7 (8.1)	41/11.0 (9.6)	87/8.7 (9.1)
Day 4 (AM)	31/5.9 (5.1)	36/10.4 (8.0)	67/8.3 (7.1)
Day 4 (PM)	23/5.2 (4.3)	29/9.3 (8.8)	52/7.5 (7.4)
Day 5 (AM)	13/6.9 (5.8)	16/11.8 (7.6)	29/9.6 (7.2)
Day 5 (PM)	6/7.8 (3.2)	14/9.9 (6.3)	20/9.2 (5.6)
Day 6 (AM)	4/7.0 (3.4)	11/9.4 (7.7)	15/8.8 (6.8)
Day 6 (PM)	2/8.5 (3.5)	9/11.8 (9.5)	11/11.2 (8.7)
Day 7 (AM)	2/4.0 (4.2)	8/9.4 (7.6)	10/8.3 (7.2)
Day 7 (PM)	1/14.0 (–)	6/11.7 (9.7)	7/12.0 (8.8)

ACSA total score range 0–64. Data are observed sample *n*/mean (SD) for participants with available data at each timepoint. Baseline at 9am on Day 1 (admission day); 1 hour at 10am on Day 1; subsequent AM assessments at 10am, PM assessments at 4pm. Rows for timepoints outside the primary analysis window (shaded) are presented for completeness but should be interpreted with caution given sparse coverage; inferential conclusions are drawn from the linear mixed model (**Table 5**), which uses the 1,236 observations within the primary analysis window under a missing-at-random assumption conditional on observed baseline covariates. Mean ± SEM error bars for **Figures 1a, b** are computed as SD/√*n* from these values. SD, standard deviation; SEM, standard error of the mean.

**Table 4:** Change in individual ACSA withdrawal symptom items from baseline to 1 hour post-device application, by user group.

ACSA symptom item	Stimulant-only users (n=102)				Stimulant/opioid co-users (n=106)			
	Baseline M (SD)	1-h M (SD)	Δ M	*p*	Baseline M (SD)	1-h M (SD)	Δ M	*p*
1. Difficulty concentrating	1.55 (1.11)	0.76 (1.10)	−0.78	<0.001	1.90 (1.41)	1.14 (1.22)	−0.75	<0.001
2. Sleeping (or wanting to sleep) a lot	2.51 (1.33)	1.81 (1.49)	−0.70	<0.001	2.83 (1.28)	2.50 (1.43)	−0.33	0.016
3. Feeling tense	1.50 (1.22)	0.67 (1.05)	−0.83	<0.001	2.13 (1.37)	1.27 (1.26)	−0.86	<0.001
4. Vivid, unpleasant dreams	1.07 (1.44)	0.52 (1.15)	−0.55	<0.001	1.19 (1.47)	0.56 (1.13)	−0.63	<0.001
5. Irritability	1.49 (1.25)	0.57 (0.99)	−0.92	<0.001	2.12 (1.33)	1.25 (1.32)	−0.88	<0.001
6. Tiredness	2.75 (1.17)	1.86 (1.47)	−0.88	<0.001	2.98 (1.16)	2.55 (1.38)	−0.43	0.001
7. Agitation	1.40 (1.26)	0.60 (1.06)	−0.80	<0.001	2.23 (1.33)	1.14 (1.31)	−1.08	<0.001
8. Feeling life is not worth living	0.32 (0.80)	0.15 (0.50)	−0.18	0.007	0.65 (1.06)	0.38 (0.80)	−0.27	0.001
9. Activity level compared to usual †	1.09 (1.22)	1.03 (1.16)	−0.06	0.451	1.08 (1.32)	1.05 (1.19)	−0.03	0.873
10. Anxiety	1.88 (1.32)	0.71 (0.98)	−1.18	<0.001	2.49 (1.28)	1.54 (1.46)	−0.95	<0.001
11. Loss of interest or pleasure	1.38 (1.48)	0.78 (1.26)	−0.60	<0.001	1.62 (1.57)	1.15 (1.32)	−0.47	<0.001
12. Difficulty trusting other people	1.96 (1.48)	1.49 (1.53)	−0.47	<0.001	1.83 (1.53)	1.29 (1.41)	−0.54	<0.001
13. Sadness	1.73 (1.37)	0.95 (1.26)	−0.77	<0.001	2.15 (1.39)	1.36 (1.36)	−0.79	<0.001
14. Feeling of slowed movements	1.25 (1.35)	0.61 (1.07)	−0.64	<0.001	1.80 (1.50)	1.27 (1.36)	−0.53	<0.001
15. Time spent craving amphetamines	1.80 (1.38)	0.65 (1.06)	−1.16	<0.001	1.58 (1.56)	0.92 (1.48)	−0.66	<0.001
16. Strength of craving for amphetamine	1.79 (1.42)	0.65 (1.13)	−1.15	<0.001	1.65 (1.62)	0.94 (1.36)	−0.71	<0.001

ACSA, Amphetamine Cessation Symptom Assessment (items scored 0 = not at all to 4 = extremely). Values are means (SD) of per-item scores at baseline (pre-device) and 1 hour post-device application, restricted to participants with a recorded value at both time points for that item; Δ M, mean within-person change, computed from unrounded per-participant differences and therefore not always equal to the difference between the two rounded column means. p values are from two-sided Wilcoxon signed-rank tests of the paired within-person change, with zero differences excluded. With a Bonferroni correction for 16 comparisons (α = 0.003), all items except item 9 remain significant in both groups, save item 8 in stimulant-only users (p = 0.007) and item 2 in co-users (p = 0.006). † Item 9 asks about activity level relative to usual and did not change significantly in either group.

### LMM: ACSA time course over the primary analysis window (baseline to day 4)

The acute device stimulation effect (post_device indicator) was significant in both the stimulant/opioid co-user and stimulant-only groups. In the stimulant-only group, the acute effect (i.e. sum of the main effect and the post_device × group interaction) was a model-implied 44.0% reduction from baseline. In the co-user reference group, the acute effect was a model-implied 30.9% reduction in ACSA score at 1 hour post-stimulation relative to baseline. The group difference in acute response was not significant.

Post-acute ACSA scores continued to decline on a decelerating log-time trajectory in both groups. In the co-user reference group, the log-time slope was significant. In the stimulant-only group, the slope was −1.33 (sum of the main slope and the log-time × group interaction). The log-time by group interaction was not significant, indicating that the post-acute rate of decrease in withdrawal symptom severity did not differ statistically between groups. The group main effect was significant: stimulant-only users scored about 4.7 points lower than stimulant/opioid co-users at baseline, a difference that persisted throughout the primary analysis window.

**Table 5** presents full fixed and random effects, with standardized estimates and semi-partial R² for each fixed effect. The acute device effect corresponded to a standardized reduction of 0.87 baseline SD units in stimulant/opioid co-users and 1.03 baseline SD units in stimulant-only users. The acute effect and the post-acute log-time slope each accounted for approximately 8% of the variance in ACSA scores (semi-partial R²=.083 and.082), and the baseline group difference for approximately 5% (.051); neither interaction term contributed appreciably. Overall model fit, computed following Nakagawa and Schielzeth (33), was R²marginal=0.30 for the fixed effects alone and R²conditional=0.79 including participant random effects.

**Table 5:** Linear mixed model fixed and random effects for ACSA total score (range 0–64) over the primary analysis window, baseline to day 4 (N = 221 participants, 1,236 observations).

Parameter	B	SE	*z*	*p*	95% CI	Std. B	sr²
Fixed effects							
Intercept (Stim+Opioid at baseline)	30.37	1.01	30.11	<.001	[28.40, 32.35]	-–	-–
Post-device indicator (acute effect)	−10.36	0.70	−14.77	<.001	[−11.74, −8.99]	−0.84	.083
Group (Stim-only vs. Stim+Opioid)	−4.72	1.44	−3.29	.001	[−7.54, −1.91]	−0.38	.051
Post-device × Group	−1.83	1.00	−1.84	.066	[−3.79, 0.12]	−0.15	.001
Log-time slope (post-acute decline)	−1.39	0.23	−5.99	<.001	[−1.85, −0.94]	−0.11	.082
Log-time × Group	0.07	0.33	0.21	.837	[−0.57, 0.70]	0.01	<.001
Random effects	Variance						
Intercept variance	81.58						
Slope variance (log-time)	2.83						
Intercept–slope covariance	−6.72						
Residual variance	32.42						

Two-phase specification with acute-effect indicator.

Model: ACSA ~ post_device × group + log_*t* × group + (1 + log_*t* | participant), where post_device is an indicator (0 at baseline, 1 at all post-baseline timepoints) representing the acute device activation effect, and log_*t* is log(hours_since_device + 0.5), set to 0 at baseline. Group coded as stimulant-only (1) vs. stimulant/opioid co-user (0, reference). Estimated by REML. The two-phase specification was selected over a single-log-time model on both mechanistic grounds (baseline-to-1-hour is a discrete device activation event) and fit grounds; a sensitivity analysis adding an AM/PM time-of-day indicator did not improve fit (likelihood-ratio χ²[1]=3.02, *p* = .082). Software: Python statsmodels 0.14. B, unstandardized coefficient; SE, standard error; CI, confidence interval; Std. B, coefficient divided by the standard deviation of ACSA across all modelled observations (12.30); sr², semi-partial R², the reduction in marginal R² when that term is removed from the model. Standardized estimates and semi-partial R² are not defined for the intercept.

**Figures 1a, b** present the observed ACSA trajectories and two-phase LMM predictions by group. **Figure 1a** shows the trajectory across the primary analysis window and the exploratory full-window fit as a dashed extension, with reference curves from McGregor et al. (19) extending to day 10; **Figure 1b** shows the first 25 hours in detail to display the acute device activation effect. Available sample sizes declined markedly beyond the primary analysis window, consistent with the real-world setting and variable duration of patient-controlled stimulation. The LMM trajectory, estimated from all 1,236 available observations, is overlaid alongside the McGregor et al. (19) medication arms as an informal visual reference. The two-phase model captures both the discrete acute reduction between baseline and 1 hour (a step of approximately 10–12 points depending on group) and the subsequent decelerating decline, in which the steepest continued reduction occurred within the first 24 hours post-stimulation with progressively smaller reductions thereafter.

**Figure 1:**
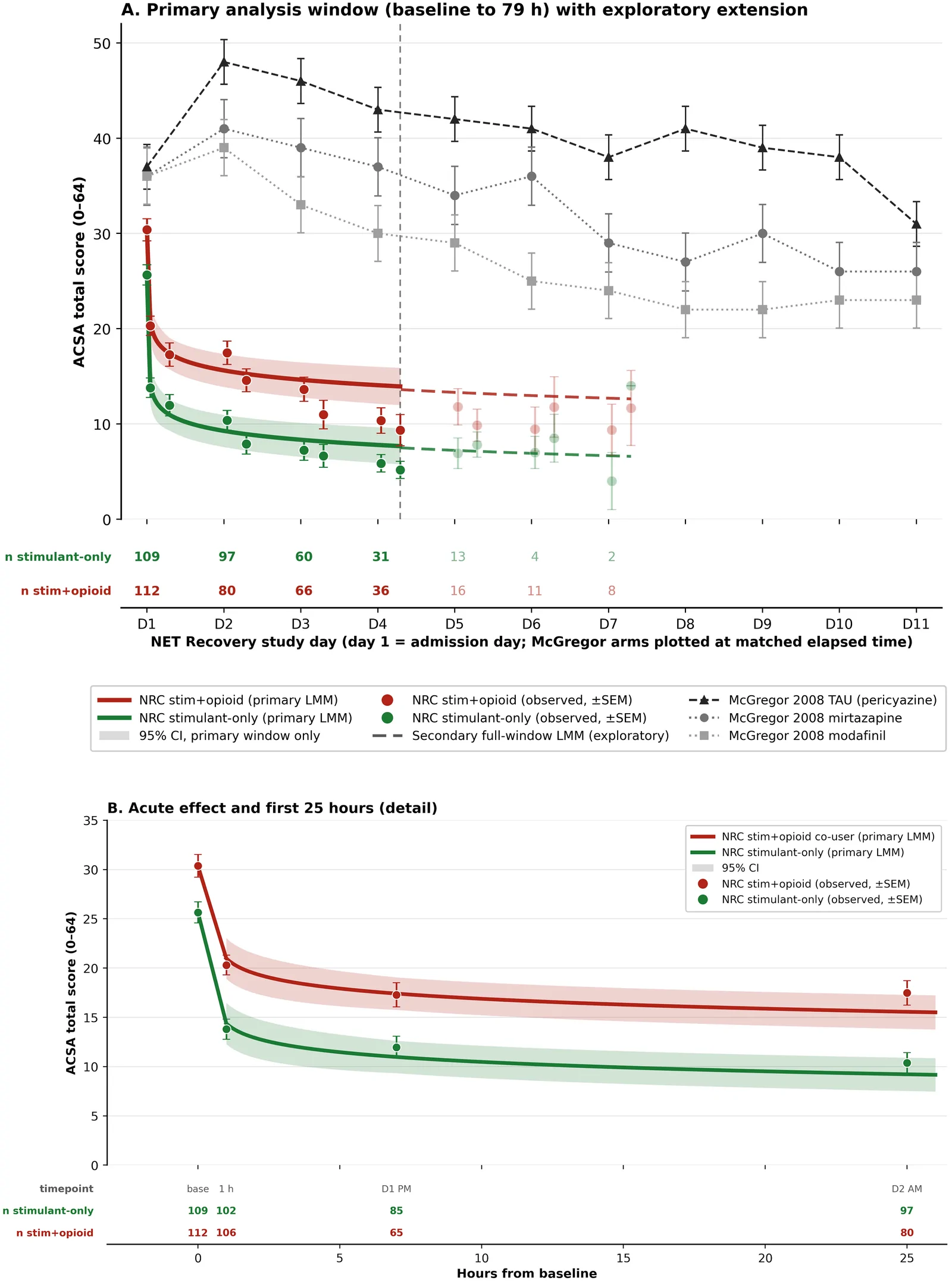
**(A)** Observed ACSA total scores (mean ± SEM) and two-phase linear mixed model-predicted trajectories, by group, across the primary analysis window (baseline to the day 4 afternoon assessment, 79 hours), with the exploratory full-window fit shown as a dashed extension to day 7 and reference curves from McGregor et al., 2008b (ref. 19) extending to day 10. **(B)** First 25 hours (detail), showing the acute device activation effect between baseline and 1 hour post-stimulation in both groups. Solid colored lines are linear mixed model point estimates; filled markers show observed group means at each timepoint (opacity reduced at timepoints outside the primary analysis window). The two-phase LMM reports acute effects (baseline to 1 hour) as a discrete indicator and post-acute decline as a log-time slope; both groups’ predicted trajectories are shown. Shaded bands are 95% confidence intervals on the fixed-effects prediction and are plotted only within the primary analysis window; dashed segments beyond 79 hours are the fitted secondary full-window model rather than an extrapolation of the primary model. No confidence band is plotted beyond the primary window because the fixed-effects interval expresses parameter uncertainty conditional on the model and widens only from ±1.9 points at day 4 to ±2.1 points at day 7 while the contributing sample falls from 69 to 7, so a band in that region would convey precision the data do not support. Attrition is instead shown directly by the numbers contributing at each timepoint beneath the axis and by the observed-mean SEM bars, which widen from ±1.1 at baseline to ±3.9 at day 7 in the stimulant/opioid co-user group. Gray scale curves (TAU pericyazine dark, mirtazapine medium, modafinil light, with distinct line styles) show the three treatment arms adapted from McGregor et al., 2008b (ref. 19); error bars on the McGregor study points are approximate SEMs based on published sample sizes (TAU *n* = 22, mirtazapine *n* = 13, modafinil *n* = 14) and typical ACSA variability. Both studies used the ACSA with scoring range 0–64; McGregor study values were digitized from image of the source paper and cross-checked against the aggregate 10-day means reported in that paper (TAU 40.9, mirtazapine 33.7, modafinil 29.7; digitization reproduces these within 1 point). Sample SDs for each NET Device timepoint are reported separately in **Table 3**. Both series are plotted against elapsed hours from each study’s own baseline; note that McGregor et al. index the admission day as day 0 whereas the present study indexes it as day 1, so day labels are offset by one between the two studies. LMM, linear mixed model; SEM, standard error of the mean; TAU, treatment as usual.

Overall, the sensitivity analysis supports the primary analysis in that significant effects of time and group, but not time by group, were observed such that the effect of the device on withdrawal symptoms was a reduction in symptoms that decreased over time in both stimulant only users and stimulant/opioid co-users (see **Supplementary Materials**).

### Comparison to published pharmacological data

In McGregor et al. (19), mirtazapine and modafinil produced meaningful ACSA reductions (relative to pericyazine, their treatment-as-usual comparator), but those reductions were not evident until days 7–10 of a 10-day inpatient stay. In the present study, comparable or greater reductions were observed by day 1 of NET Device use. Baseline ACSA scores in the present sample were lower than those reported by McGregor et al. (19), likely reflecting differences in sample composition and severity at admission. A cross-study statistical comparison was not conducted due to substantial differences in methodology and context.

### Safety

No device-related adverse events were reported across the 221 participants during the treatment period.

### Sensitivity analysis for the MAR assumption

The primary findings were tested against plausible departures from the MAR assumption using a pattern-mixture sensitivity analysis. Intuitively, the analysis asks: if participants who stopped reporting actually had worse (higher) ACSA scores than our MAR-based predictions assume, at what point would the results change? For each missing post-baseline ACSA value, we imputed a value equal to the MAR-based prediction (individualized by each participant’s random effects) plus a fixed shift (delta) and residual noise. A positive delta represents failure-driven MNAR: it assumes unobserved scores are delta points higher than MAR predicts, every time. Ten imputations were generated for each delta ∈ {0, 5, 10, 15, 20}; the LMM was refit on each completed dataset and results pooled via Rubin’s rules. At delta=0, pooled estimates reproduced the primary MAR fit, validating the procedure. The acute device effect (post_device coefficient) remained highly significant and stable in magnitude across the full delta range (B=−10.41 at delta=0 to B=−8.17 at delta=20; all *p*s<.001); the baseline group difference and the non-significant log-time × group interaction were similarly unaffected. The post-acute log-time slope was the sensitive parameter: significant at delta=0 (B=−1.37, *p* <.001) and delta=5 (B=−0.83, *p* <.001), non-significant at delta=10 (B=−0.28, *p* = .225), and reversed in sign at delta=15 and 20. The tipping point for the log-time slope thus lies at approximately delta=10, corresponding to a scenario in which typical participants who ended stimulation early would have had ACSA scores roughly 1.8 residual SDs higher than MAR predicts at every post-termination timepoint, approaching their baseline severity. Such a scenario is inconsistent with the documented 40–60% natural resolution of stimulant withdrawal over 7–10 days (9, 10) and is considered clinically implausible. The primary findings are therefore robust to plausible MNAR departures. Device use duration was also tested as a moderator of the post-acute log-time slope and showed no significant effect (duration × log-time B=−0.02 per 10 additional hours, SE 0.05, *p* = .74; likelihood-ratio χ²[1]=0.11, *p* = .74).

## Discussion

### Principal findings

This real-world data (RWD) analysis provides the first prospective evidence, generated under routine open-label clinical conditions (i.e. without sham control), that NET Device monotherapy is associated with rapid and substantial reduction in psychostimulant withdrawal severity. The two-phase LMM, applied to all 1,236 observations within the primary analysis window under a missing-at-random framework, quantified two components of the response: an acute ACSA reduction of approximately 10–12 points (32–44%) between baseline and 1 hour post-device stimulation, and a continuing decelerating decline on a log-time trajectory through day 4 (79 hours) with statistically equivalent rates across groups. Stimulant-only users reported less-severe withdrawal (about 4.4 points lower) than stimulant/opioid co-users at baseline and this difference persisted across the observation window. Observed ACSA scores were approximately 46% lower at 1 hour and 59% lower by day 2 morning in stimulant-only users; and 33% and 43% lower at the same timepoints in stimulant/opioid co-users. These reductions were substantially faster than the natural resolution of stimulant withdrawal, previously reported as 40–60% over 7–10 days (14, 15), but interpretation must be tempered by dropout during the observation window.

Rapid reduction of stimulant withdrawal symptom severity is clinically important. The early period (first days) of stimulant discontinuation is when patients are most symptomatic, distressed, and at highest risk for treatment dropout and return to use. An intervention that accelerates withdrawal suppression from the second week of treatment (medications) into the first 24 hours (NET Device) directly addresses the engagement barrier at the most critical clinical juncture. While this RWD analysis cannot establish whether faster withdrawal resolution translates to better treatment retention or longer-term outcomes, and is primarily limited to the first 4 days (due to high attrition thereafter), the mechanistic pathway is plausible: rapidly reducing physiological burden during the earliest phase of care may lower barriers to engaging with subsequent behavioral interventions (21).

The difference in ACSA trajectory between stimulant-only and stimulant/opioid co-users is notable. Co-users presented with higher baseline ACSA scores, reflecting greater aggregate symptom burden. Both groups showed substantial acute reductions, but stimulant-only users showed numerically greater relief at early timepoints. This group difference could occur for several reasons: (i) partial ACSA overlap with opioid withdrawal symptoms, thereby attenuating apparent stimulant-specific improvement in the co-using group; (ii) greater physiological complexity requiring more time to resolve; or (iii) the device stimulation waveforms were programmed on the first day to address opioid withdrawal symptoms, then on the second day waveforms were added to address stimulant withdrawal symptoms, which (despite stimulant withdrawal symptoms often emerging later than opioid withdrawal and being less disruptive) could have led to overall greater ACSA scores than the stimulant-only group. The equivalence of outcomes across residential and detention settings is notable, particularly given the substantial differences in standard-of-care delivery between these environments (structured psychosocial programming in residential treatment vs. minimal behavioral support in detention), and supports deployment across diverse clinical environments.

### Potential mechanisms of action

The mechanisms of action for this device-related stimulant withdrawal suppression effect remain to be determined at several levels of analysis. First, we provide background on this form of neuromodulation. tACS applies alternating currents (typically in the range of 1 to 3.4 mA) which produce weak electrical fields (≤1 V/m) that stimulate cortical layer V pyramidal cells which are primarily excitatory glutamatergic. In addition to electrical current intensity, the orientation and frequency of tACS perturbs neuronal membrane potential, which can modulate the probability (but not rate) of action potentials (34, 35). tACS has been theorized, mathematically modeled, and shown electrophysiologically to promote entrainment (phase-locking) of cortical network rhythms (36, 37). This entrainment is strongest at the driving frequency of stimulation (but also its harmonic and sub-harmonic multiples, which introduces cross-frequency interactions such as gamma-frequency inhibition of alpha power; e.g (38). Neural synchronization occurs primarily in the cortical region underlying scalp stimulation (39–41), but may propagate to and couple with other brain regions via cortico-cortical connections (42–44). Although less well studied, some findings suggest that, under some conditions, tACS may produce effects that extend beyond cessation of stimulation, distinct from entrainment, that may be due to several processes including spike-dependent plasticity, intracellular calcium dynamics, and protein synthesis (45–50).

How might these tACS electrophysiological mechanisms relate to stimulant withdrawal symptoms? Evidence from animal studies suggests that stimulant withdrawal following chronic exposure leads to region-specific, hypofunctional dopamine signaling-mediated decreases in intrinsic neuronal excitability and basal activity, via lower sodium (Na^+^) and calcium (Ca^2+^) channel conductance, and higher potassium (K^+^) channel conductance (51–53). Withdrawal following chronic stimulant exposure has also been shown to increase GABA relative to glutamate signaling (54, 55) and to upregulate cholinergic (56, 57), corticotropin releasing factor (58, 59), and dynorphin (60, 61) systems, while downregulating serotonin (62, 63). Taken together, these and other neurophysiological alterations may be associated with the constellation of withdrawal symptoms (e.g. anhedonia, depression, anxiety, irritability, poor concentration) and risk of recurrent use (64, 65).

We propose that tACS during the period of stimulant withdrawal could increase neuronal membrane excitability and associated dysregulations in dopamine, glutamate/GABA, and other systems, re-tuning these brain systems to be more sensitive to environmental inputs such as natural reinforcers including supportive therapeutic interventions. Although the individual is also likely to be more sensitive to drug re-exposure effects during this same period, device use occurs in a controlled setting, which may establish context-dependent learning between neurobiological changes and naturalistic reinforcement. Furthermore, given that many participants self-titrate their NET Device use over a long period (averaging 40 hours in this sample, a substantial epoch length compared to experimental studies), we hypothesize that NET Device use at the programmed frequencies has significant potential to induce not only short-term entrainment of cortical rhythms but also longer-term neuroplasticity. In future studies, we plan to incorporate biomarkers of these systems to improve our understanding of the mechanistic basis of NET Device effects (and tACS more broadly).

### Absence of approved treatment: clinical and regulatory context

The present data contribute to a clinical and regulatory landscape in which stimulant use disorder lacks any FDA-approved pharmacotherapy. This gap is not for lack of investigative effort: over the past 30 years, dozens of randomized controlled trials have evaluated antidepressants, antipsychotics, anticonvulsants, dopamine agonists, and stimulants themselves; systematic reviews consistently conclude that no agent has demonstrated sufficient efficacy, safety, and replicability to warrant regulatory approval (5–9). For the acute stimulant withdrawal phase specifically, reviews have found no significant pharmacological advantage over placebo (6, 16–18). The NET Device, as a non-pharmacological approach evaluated under a device regulatory framework, represents a distinct investigational pathway for addressing this clinical gap.

Other neuromodulation approaches are emerging as potential treatments for stimulant use disorder. In recent reviews, repetitive transcranial magnetic stimulation (rTMS), specifically high-frequency stimulation (10 Hz or intermittent theta burst) over the left dorsolateral prefrontal cortex (dlPFC) was concluded to be effective for reducing craving (66–72). However, neuromodulation studies have not typically focused on reducing stimulant withdrawal symptoms. To our knowledge, only one randomized, sham-controlled trial has been conducted, which found that active dlPFC rTMS was effective in reducing withdrawal symptoms in male methamphetamine users (73). While initial findings are promising, none of these therapies has received FDA marketing clearance for stimulant use disorder. Notably, these trials have not examined mechanisms of action, so it is presently unclear whether there is overlap between the neurobiological effects of high-frequency dlPFC rTMS, tDCS, and the NET Device.

The present dataset constitutes real-world evidence (RWE) within the FDA framework: data collected prospectively from routine clinical operations, across multiple facility types and geographies, outside a controlled experimental design. At *N* = 221, it is the largest prospective dataset evaluating NET Device effects on stimulant withdrawal to date. It complements the pivotal sham-controlled trial for opioid withdrawal suppression (24), in which the active arm experienced a mean 61.3% COWS reduction at 1 hour post-stimulation and 98.1% achieved the primary endpoint of ≥15% COWS reduction. The pivotal trial did not measure ACSA, so a direct cross-study comparison on stimulant-specific symptoms is not possible. However, half of the pivotal trial sample screened positive for psychostimulants (primarily methamphetamine) at intake, enabling an informative subgroup analysis of opioid withdrawal response within the stimulant co-using population. In a *post-hoc* subgroup analysis of the pivotal trial dataset, all 25 active-arm participants who were stimulant-positive at baseline achieved the ≥15% COWS reduction endpoint at 1 hour (25/25, 100%), with a mean COWS reduction of 63.5% (SD 19.4%); the corresponding sham-arm stimulant-positive subgroup (*n* = 28) showed 46.5% mean reduction and 85.7% ≥15% responders (active vs. sham within the stimulant-positive subgroup: Welch’s *t* = 2.41, *p* = .020; **Supplementary Table 6**). Thus, in the stimulant co-using population of the pivotal opioid-withdrawal trial, active NET Device use produced acute withdrawal reductions on COWS that are similar in magnitude to the acute ACSA reductions observed in the present stimulant-focused RWD dataset. The convergence of a controlled trial (pivotal study, stimulant-positive subgroup) and independent RWE (present study) across different study designs, populations, withdrawal instruments, and settings strengthens the evidentiary foundation for a planned confirmatory trial of the NET Device in stimulant use disorder.

### Limitations

One significant limitation is the absence of a sham or control condition. RWD analyses cannot distinguish device-specific effects from natural withdrawal resolution, expectancy, non-specific treatment engagement effects, or concurrent adjunctive care. The observed ACSA reductions may overestimate the specific device contribution. A second limitation is that individuals who received access to the device were not randomly selected, and several baseline factors (e.g., race, desire to avoid medications, individual differences in stimulant use disorder severity) could confound the present findings and limit generalizability. Specifically, the sample was drawn from residential and detention facilities in rural Kentucky and was predominantly white; participants who received the device may have been more tolerant/accepting of non-pharmacological approaches and/or less interested in medications; and baseline differences in ACSA scores were associated with duration of device use and number of observations. A third limitation is that participant inclusion was based on stimulant-positive UDS and/or past-month reported use of stimulants, rather than a formal diagnosis of Stimulant Use Disorder. Specifically, we were unable to obtain substance use disorder diagnoses in detention centers, where routine assessments focused on medical history and physical exam, and substance use history but not diagnoses. Future research should examine diagnosis and severity to determine whether these could play a role in the findings. This may be important because our sample was likely less physically dependent on stimulants than patients in the McGregor et al. study (19). Fourth, participants drawn from these addiction treatment facilities and detention centers received heterogeneous types of clinical care, and care in detention centers was generally more limited. This could potentially affect findings, although we tested for and did not observe differences in device use or symptom outcomes between these settings. Additionally, the RWD offered an opportunity to explore effects in the context of heterogenous clinical care, thus making the findings more applicable to common care contexts in which care is likely to be heterogeneous. A fifth limitation is that many participants ended device stimulation before day 7. Missing data were handled under a missing-at-random (MAR) assumption, supported by the observed relationship between early end-of-stimulation and baseline severity and by the mechanistic account of success- and failure-driven end of stimulation (see Statistical Analysis section). MAR cannot be verified from the data alone. A pattern-mixture sensitivity analysis showed that the primary acute-effect finding is robust across a wide range of MNAR departures, and the post-acute log-time slope is robust up to moderate MNAR assumptions. Because of substantial attrition after day 4, the primary analysis window was restricted to baseline through the day 4 afternoon assessment (79 hours, encompassing 93.1% of observations). Descriptive means and model estimates for day 5 onward are reported as exploratory and are not the basis for any efficacy claim. A sixth limitation is that per-participant assessment timestamps were not available; elapsed time was assigned from the protocol-specified uniform facility schedule, so any departure from that schedule introduces error in the time covariate. Seventh, device use duration varied widely, and no minimum threshold was applied; participants with very short utilization may not have received meaningful stimulation. We did not collect self-report data on specific reasons for stoppage of device use. These and potentially other limitations are characteristic of RWD analyses, preclude causal attribution, and will be directly addressed by a planned sham-controlled randomized trial.

## Conclusion

In this RWD analysis of 221 stimulant-positive adults entering residential addiction treatment or detention in Kentucky, NET Device monotherapy was associated with rapid and marked reduction in psychostimulant withdrawal severity. ACSA scores decreased 34–47% from baseline at 1 hour post-device activation, followed by a decelerating decline. Stimulant/opioid co-users (with higher absolute scores overall) stimulant-only users both showed substantial improvement. No device-related adverse events were reported. The rapidity of withdrawal reduction substantially exceeded the pace of natural resolution. As RWE generated under routine clinical conditions (with limitation as noted including no sham control and substantial attrition) and consistent with prior sham-controlled pivotal trial results for opioid withdrawal suppression, these novel findings support the initiation of a sham-controlled randomized trial and contribute to the regulatory evidence base for the NET Device in stimulant use disorder.

## Data Availability

The datasets presented in this article are not readily available due to patient confidentiality issues. Requests for data required to support the protocol will be evaluated by the sponsor and provided if deemed appropriate. All study-related information will be regarded as confidential. Requests to access the data should be directed to the corresponding author.
