# Supplementary material for: Effect of transcranial alternating current stimulation (NET Device™) on psychostimulant withdrawal severity and time course: a real-world data analysis
Source: Front Psychiatry. 2026 Sep 24;17:1899599. doi: 10.3389/fpsyt.2026.1899599 (PMC13647114; doi:10.3389/fpsyt.2026.1899599)
Supplement: Supplementary file 1 [file SupplementaryFile1.docx]

**SUPPLEMENTARY MATERIALS**

**Supplementary Methods**

***Primary linear mixed model (LMM) statistical analysis of ACSA scores***

Formally, ACSA_ij = β0 + β1·post_device_ij + β2·log_t_ij + β3·group_i + β4·(post_device × group) + β5·(log_t × group) + u_0i + u_1i·log_t_ij + e_ij, where i indexes participant and j indexes timepoint; post_device_ij is the acute-effect indicator (0 at baseline, 1 post-baseline); log_t_ij = log(hours_since_device + 0.5), set to 0 at baseline; group_i is the stimulant-only vs. stimulant/opioid co-user contrast (1 vs. 0); u_0i and u_1i are participant-specific random intercept and random slope on log_t; and e_ij is the residual.

***Supplementary statistical analysis***

Supplementary statistical analyses were performed by an additional biostatistician not privy to the primary analyses.

Alternative linear mixed effects sensitivity analysis. To determine whether the primary findings were specific to the two-phase model specification or generally represented the phenomena of interest—that is, a persistent decrease in withdrawal severity four days post stimulation—we iteratively fit linear mixed effects models testing (i) the fixed effects of time (coded 0-8; see below), (ii) group (coded 0 for stimulant-only users and 1 for stimulant/opioid co-users), and (iii) two-way interactions between group and time. While data were collected up to 7 days post stimulation, morning and evening datapoints from days 5-7 were excluded from models due to abundance of missing data (87.1%-96.97% missing). Models tested participant-specific random intercepts only, random intercepts and random slopes, and the additional nested structure of setting (residential vs. detention).

As with the primary analysis, the models used all 1,266 observations from 221 participants estimated by restricted maximum likelihood estimation. Sensitivity analysis was conducted in R version 4.6.0 running on top of RStudio.

Rationale for modeling time as a continuous variable. In this alternative approach, we treat time as a continuous variable to examine the relation between time and psychostimulant withdrawal severity. By representing time as continuous events from baseline (‘0’), 1 hour post-stimulation (‘1’), the subsequent afternoon assessment (‘2’), and through 4 days post-stimulation at morning and afternoon assessment points (‘3’ – ‘8’), we are able to model the strength and shape of the effect of stimulation is on psychostimulant withdrawal severity over time. Compared to the design of the two-phase model from our primary analysis, this more parsimonious approach assesses change over time rather than discrete timepoints when change occurred (e.g., from device-off [baseline] to device-on [1 hour post-stimulation]).

**Results**

***Justification for linear mixed effects models***

A null model indicated that the intercept differed from 0 (B=16.57, *t*(205.41)=25.13, *p*<.001) and provided evidence for random effects by participant with an ICC value of 0.49.

***Decrease in withdrawal symptoms over time***

The random intercepts only model indicated significant effects of time and group, but not time by group (see **Supplementary Table 1**).

**Supplementary Table 1.** Random intercepts only model. Model fit was significant improved as compared to the null model, see **Supplementary Table 4.**

|  | **Fixed Effects** | | | | |
| --- | --- | --- | --- | --- | --- |
|  | **Standardized Est.** | ***S.E.*** | ***t*** | ***df*** | ***p*** |
| *Intercept* | 19.73 | 0.91 | 21.79 | 296.62 | <.001 |
| *Time* | -0.45 | 0.14 | -17.51 | 1095.62 | <.001 |
| *Group* | 0.26 | 1.27 | 5.03 | 298.70 | <.001 |
| *Time by Group* | -0.01 | 0.19 | -0.22 | 1107.85 | 0.820 |
|  | **Random Effects** | | | | |
|  | **Parameter** | ***SD*** |  |  |  |
| *Participant* | Intercept | 8.06 |  |  |  |
| *Residual* |  | 7.16 |  |  |  |
|  | ***ICC*** |  |  |  |  |
| *Participant* | 0.56 |  |  |  |  |

Note: Intercept estimates are unstandardized.

Participants who used stimulants and opioids reported greater withdrawal severity compared to participants who used stimulants alone, however the change in withdrawal severity did not differ by use groups (see **Supplementary Figure 1**).

**Supplementary Figure 1.** Change in withdrawal severity over time, faceted by substance use group. Shading around the linear fit line represents standard error.

A random slopes term was then added to the model, allowing the effect of time to randomly vary by participant (see **Supplementary Figure 2**).

**Supplementary Figure 2**. Variability in change over time by participant. SID reflects the participant identifier color-coding each point and line. Participant data is faceted by substance use group.

The random intercepts, random slopes model indicated significant effects of time and group, but not time by group (see **Supplementary Table 2**).

**Supplementary Table 2.** Random intercepts, random slopes model. Model fit was significant improved as compared to the random intercepts only model, see **Supplementary Table 4**.

|  | **Fixed Effects** | | | | |
| --- | --- | --- | --- | --- | --- |
|  | **Standardized Est.** | ***S.E.*** | ***t*** | ***df*** | ***p*** |
| *Intercept* | 20.26 | 1.01 | 20.07 | 216.72 | <.001 |
| *Time* | -0.51 | 0.20 | -13.67 | 118.97 | <.001 |
| *Group* | 0.24 | 1.42 | 4.20 | 217.45 | <.001 |
| *Time by Group* | 0.04 | 0.28 | 0.72 | 113.85 | 0.480 |
|  | **Random Effects** | | | | |
|  | **Parameter** | ***SD*** |  |  |  |
| *Participant* | Intercept | 9.58 |  |  |  |
| *Participant* | Time | 1.43 |  |  |  |
| *Residual* |  | 6.47 |  |  |  |
|  | ***ICC*** |  |  |  |  |
| *Participant* | 0.68 |  |  |  |  |

Note: Intercept estimates are unstandardized.

We added an additional nested level—facility type—to the best-fitting random intercepts, random slopes model to account for the potential non-independence of observations within facility types, such that the mean and change in severity of withdrawal may be shared within facility types. There was no supporting evidence for non-independence of observations within facility types (see **Supplementary Table 3**), and the inclusion of this additional nested factor did not improve model fit (see **Supplementary Table 4**). These findings are aligned with the lack of differences in ACSA severity and time course between residential treatment and detention center subgroups (see **Supplementary Figure 3**) that motivated combination of setting groups for primary analyses.

**Table 3.** Random intercepts, random slopes model with additional nested structure by facility type (residential treatment or detention center). The addition of this second nested factor did not significantly improve model fit, see **Supplementary Table 4.**

|  | **Fixed Effects** | | | | |
| --- | --- | --- | --- | --- | --- |
|  | **Estimate** | ***S.E.*** | ***t*** | ***df*** | ***p*** |
| *Intercept* | 20.26 | 1.01 | 20.07 | 216.62 | <.001 |
| *Time* | -0.51 | 0.20 | -13.67 | 118.94 | <.001 |
| *Group* | 0.24 | 1.42 | 4.20 | 217.35 | <.001 |
| *Time by Group* | 0.04 | 0.28 | 0.72 | 113.83 | 0.480 |
|  | **Random Effects** | | | | |
|  | **Parameter** | ***SD*** |  |  |  |
| *Participant* | Intercept | 5.07 |  |  |  |
| *Participant* | Time | 1.44 |  |  |  |
| *Participant by Facility Type* | Intercept | 8.01 |  |  |  |
| *Facility Type* | Intercept | 0.00 |  |  |  |
| *Residual* |  | 6.47 |  |  |  |
|  | ***ICC*** |  |  |  |  |
| *Participant* | 0.20 |  |  |  |  |
| *Participant by Facility Type* | 0.49 |  |  |  |  |
| *Facility Type* | 0.00 |  |  |  |  |

Note: Intercept estimates are unstandardized.

**Supplementary Figure 3**. Change in withdrawal severity over time, faceted by facility type. Shading around the linear fit line represents standard error.

**Supplementary Table 4.** Model fit indices and comparisons between linear mixed effects models.

|  | **Model Fit Indices** | | | |  |  |  |
| --- | --- | --- | --- | --- | --- | --- | --- |
|  | **AIC** | **BIC** | **Pseudo-R^2^ (fixed effects)** | **Pseudo-R^2^ (total)** | **Chi-Squared** | ***df*** | ***p*** |
| *Null Model* | 9560.70 | 9576.10 | 0.00 | 0.49 | - | - | - |
| *Random Intercept Only, nested by Participant* | 9036.10 | 9067.00 | 0.26 | 0.67 | 530.56 | 3 | <.001 |
| *Random Intercepts, Random Slopes, nested by Participant* | 8984.50 | 9025.70 | 0.28 | 0.75 | 55.60 | 2 | <.001 |
| *Random Intercepts, Random Slopes, nested by Participant and Facility* | 8988.50 | 9040.00 | 0.28 | 0.75 | 0.00 | 2 | 1.00 |

Finally, we tested the effects of demographic covariates (age, sex, race) in our model of best fit, the random intercepts, random slopes model nested by participant. Age, sex, and race (with multiracial and participants endorsing race as ‘Other’ removed from the model given small cell sizes) were not significant predictors of withdrawal severity (see **Supplementary Table 5**). The inclusion of covariates in the model did not improve model fit (AIC=8504.10, BIC=8560.00, Pseudo *R*^2^_fixed effects_=0.28, Pseudo *R*^2^_total effects_=0.75, *χ*^2^(3)=3.53, *p*=0.317.

**Supplementary Table 5.** *Addition of covariates to random intercepts, random slopes model, nested by participant.*

|  | **Fixed Effects** | | | | |
| --- | --- | --- | --- | --- | --- |
|  | **Standardized Est.** | ***S.E.*** | ***t*** | ***df*** | ***p*** |
| *Intercept* | 21.62 | 2.57 | 8.42 | 217.23 | <.001 |
| *Time* | -0.51 | 0.20 | -13.32 | 112.23 | <.001 |
| *Group* | 0.22 | 1.50 | 3.61 | 208.77 | <.001 |
| *Time by Group* | 0.04 | 0.28 | 0.67 | 107.59 | 0.500 |
| *Age* | 0.002 | 0.06 | 0.04 | 191.89 | 0.970 |
| *Sex (ref. = females)* | -0.09 | 1.23 | -1.83 | 194.55 | 0.070 |
| *Race (ref. = white)* | -0.02 | 2.13 | -0.30 | 184.32 | 0.770 |
|  | **Random Effects** | | | | |
|  | **Parameter** | ***SD*** |  |  |  |
| *Participant* | Intercept | 9.54 |  |  |  |
| *Participant* | Time | 1.38 |  |  |  |
| *Residual* |  | 6.48 |  |  |  |
|  | ***ICC*** |  |  |  |  |
| *Participant* | 0.68 |  |  |  |  |

Note: Intercept estimates are unstandardized.

**Supplementary Table 6.** *Post-hoc subgroup analysis of the NET Device pivotal sham-controlled trial for opioid withdrawal suppression (ref 24), by treatment arm and baseline stimulant status.*

| **Subgroup** | **N** | **Baseline COWS, mean (SD)** | **1-hour COWS, mean (SD)** | **Mean % reduction (SD)** | **Achieved ≥15% reduction** |
| --- | --- | --- | --- | --- | --- |
| **Active arm (overall)** | **52** | 18.1 (4.4) | 7.0 (4.1) | 61.0% (21.7) | 51/52 (98.1%) |
| Active arm, stimulant-positive | 25 | 19.5 (5.0) | 7.0 (3.9) | 63.5% (19.4) | 25/25 (100.0%) |
| Active arm, stimulant-negative | 27 | 16.9 (3.4) | 7.0 (4.3) | 58.7% (23.8) | 26/27 (96.3%) |
| **Sham arm (overall)** | **55** | 18.6 (4.0) | 9.8 (5.4) | 45.8% (29.9) | 46/55 (83.6%) |
| Sham arm, stimulant-positive | 28 | 18.2 (2.8) | 9.6 (5.9) | 46.5% (31.2) | 24/28 (85.7%) |
| Sham arm, stimulant-negative | 27 | 19.0 (4.9) | 10.0 (5.0) | 45.2% (29.0) | 22/27 (81.5%) |

Data are from the NRC021 intent-to-treat dataset (N=108 randomized; 52 active and 55 sham with valid 1-hour COWS; 1 active participant dropped out prior to 1-hour assessment and is excluded from this analysis). Stimulant-positive status was defined as any positive result on urine drug screen for amphetamine, methamphetamine, cocaine, or MDMA at baseline (54/108 = 50.0% of ITT sample). Overall active- and sham-arm rows (shaded) reproduce published figures from Greenwald et al. 2025 (ref 15) and are provided as validation anchors for the subgroup analysis. Within the stimulant-positive subgroup, the active-vs-sham difference in mean percent COWS reduction was statistically significant (Welch’s t=2.41, df=38.7, p=.020); the active-vs-sham difference in ≥15% responder rate approached significance (100% vs. 85.7%; Fisher’s exact p=.113). COWS, Clinical Opiate Withdrawal Scale; SD, standard deviation.
